# Development and Validation of a Simplified Prehospital Triage Model Using Neural Network to Predict Mortality in Trauma Patients: The Ability to Follow Commands, Age, Pulse Rate, Systolic Blood Pressure and Peripheral Oxygen Saturation (CAPSO) Model

**DOI:** 10.3389/fmed.2021.810195

**Published:** 2021-12-10

**Authors:** Yun Li, Lu Wang, Yuyan Liu, Yan Zhao, Yong Fan, Mengmeng Yang, Rui Yuan, Feihu Zhou, Zhengbo Zhang, Hongjun Kang

**Affiliations:** ^1^Medical School of Chinese PLA, Beijing, China; ^2^Department of Critical Care Medicine, The First Medical Centre, Chinese PLA General Hospital, Beijing, China; ^3^Center for Artificial Intelligence in Medicine, Chinese PLA General Hospital, Beijing, China

**Keywords:** trauma, in-hospital mortality, prehospital, triage, scoring system, machine learning

## Abstract

**Objective:** Most trauma scoring systems with high accuracy are difficult to use quickly in field triage, especially in the case of mass casualty events. We aimed to develop a machine learning model for trauma mortality prediction using variables easy to obtain in the prehospital setting.

**Methods:** This was a retrospective prognostic study using the National Trauma Data Bank (NTDB). Data from 2013 to 2016 were used for model training and internal testing, and data from 2017 were used for validation. A neural network model (NN-CAPSO) was developed using the ability to follow commands (whether GCS-motor was <6), age, pulse rate, systolic blood pressure (SBP) and peripheral oxygen saturation, and a new score (the CAPSO score) was developed based on logistic regression. To achieve further simplification, a neural network model with the SBP variable removed (NN-CAPO) was also developed. The discrimination ability of different models and scores was compared based on the area under the receiver operating characteristic curve (AUROC). Furthermore, a reclassification table with three defined risk groups was used to compare NN-CAPSO and other models or scores.

**Results:** The NN-CAPSO had an AUROC of 0.911(95% confidence interval 0.909 to 0.913) in the validation set, which was higher than the other trauma scores available for prehospital settings (all *p* < 0.001). The NN-CAPO and CAPSO score both reached the AUROC of 0.904 (95% confidence interval 0.902 to 0.906), and were no worse than other prehospital trauma scores. Compared with the NN-CAPO, CAPSO score, and the other trauma scores in reclassification tables, NN-CAPSO was found to more accurately classify patients to the right risk groups.

**Conclusions:** The newly developed CAPSO system simplifies the method of consciousness assessment and has the potential to accurately predict trauma patient mortality in the prehospital setting.

## Introduction

Trauma remains one of the leading causes of death and disability worldwide (1). Patients with severe trauma often benefit from receiving treatment at a higher level of care (2). Therefore, it is important to identify patients with severe trauma in the prehospital setting to avoid delayed or inadequate treatment, especially after a mass casualty incident (MCI). However, in many cases, the prehospital phase of triage is time-constrained and aids to diagnosis are limited, and even when many ambulance personnel do not have the relevant specialization, the number of personnel is severely insufficient compared to the large number of casualties (3). Thus, investigating how to quickly and accurately determine the severity of injuries using the most accessible assessment methods is needed.

To date, many severity assessment methods applicable to the early stages of trauma have been proposed and validated, including scoring systems or predictive models, most of which were constructed based on logistic regression analysis. The Revised Trauma Score (RTS), which was first proposed in 1989, used respiratory rate (RR), systolic blood pressure (SBP), and Glasgow Coma Scale (GCS) to calculate the probability of survival and is still widely used today (4). The Mechanism, Glasgow Coma Scale, Age, and Arterial Pressure (MGAP) score developed in 2010 used four variables to assess the severity of trauma and performed better than the RTS (5). In contrast, the Glasgow Coma Scale, Age, Systolic Blood Pressure (GAP) score was proposed in 2011, which was referenced for the establishment of the MGAP, performed no less well than the MGAP with the mechanism of trauma removed (6). The NTS (New Trauma Score) score used peripheral oxygen saturation (SpO_2_) instead of respiratory rate in the RTS and improved the prediction of death in trauma patients (7). The Trauma Rating Index in Age, Glasgow Coma Scale, Respiratory rate and Systolic blood pressure (TRIAGES) score used a generalized additive model to delineate the interval of variables and had better performance than the GAP score with the addition of the respiratory rate variable (8). Composed by the mechanism of trauma RTS, Injury Severity Score (ISS) (9) and age, the Trauma and Injury Severity Score (TRISS) was able to predict trauma mortality accurately (10). Although TRISS can hardly be used in prehospital settings because of the complex assessment of ISS, it is often taken as a benchmark for comparison with other trauma scores. However, in mass casualty incidents, it is also difficult to have sufficient time and manpower to monitor and assess all vital signs and complete GCS scores of casualties. Without the use of assistive electronic devices, calculating scores at the scene also has the disadvantage of being time-consuming and error-prone. In addition, the reliability of complete GCS score is dependent on relevant training and education (11), and it is often difficult for nonprofessional personnel involved in triage to accurately assess the GCS (12).

Therefore, it is necessary to explore the optimization of the input variables that need to be evaluated prehospital, for example, by considering the simplification of the consciousness assessment method (13) or by eliminating the systolic blood pressure variable, which is relatively difficult to measure (14). Alternatively, scores can be calculated quickly and accurately with the help of electronic devices, or for better prediction, sophisticated machine learning models can be embedded in them. Machine learning models can often better handle complex non-linear interactions between variables and improve the accuracy of results by optimizing the error between predicted and observed results (15). Other studies have shown that using only the motor component of the GCS is a simple and valid assessment tool, and even determining whether a patient has the ability to follow commands (assessing whether the GCS-motor is <6) has been shown to be a potential alternative to the GCS in the prehospital phase (16). However, there are no valid machine learning models or scores using this approach developed for predicting the mortality of trauma patients in the prehospital setting. The purpose of this study is to investigate the development of a machine learning model and a new easy-to-use trauma score for prehospital trauma mortality prediction. This will be achieved by using the binary assessment of GCS-motor (GCS-m) score <6 and other accessible vital signs, of which the predictive performance is not inferior to the RTS, MGAP, GAP, and TRIAGES scores.

## Methods

### Study Design and Setting

Data were obtained from the National Trauma Data Bank (NTDB), the largest trauma database in the United States, which was assembled by the American College of Surgeons (17). Reporting of this study followed the Transparent Reporting of a Multivariable Prediction Model for Individual Prognosis or Diagnosis (TRIPOD) Guideline (18). Permission to use these data was obtained from NTDB.

### Selection of Participants

**Figure 1:**
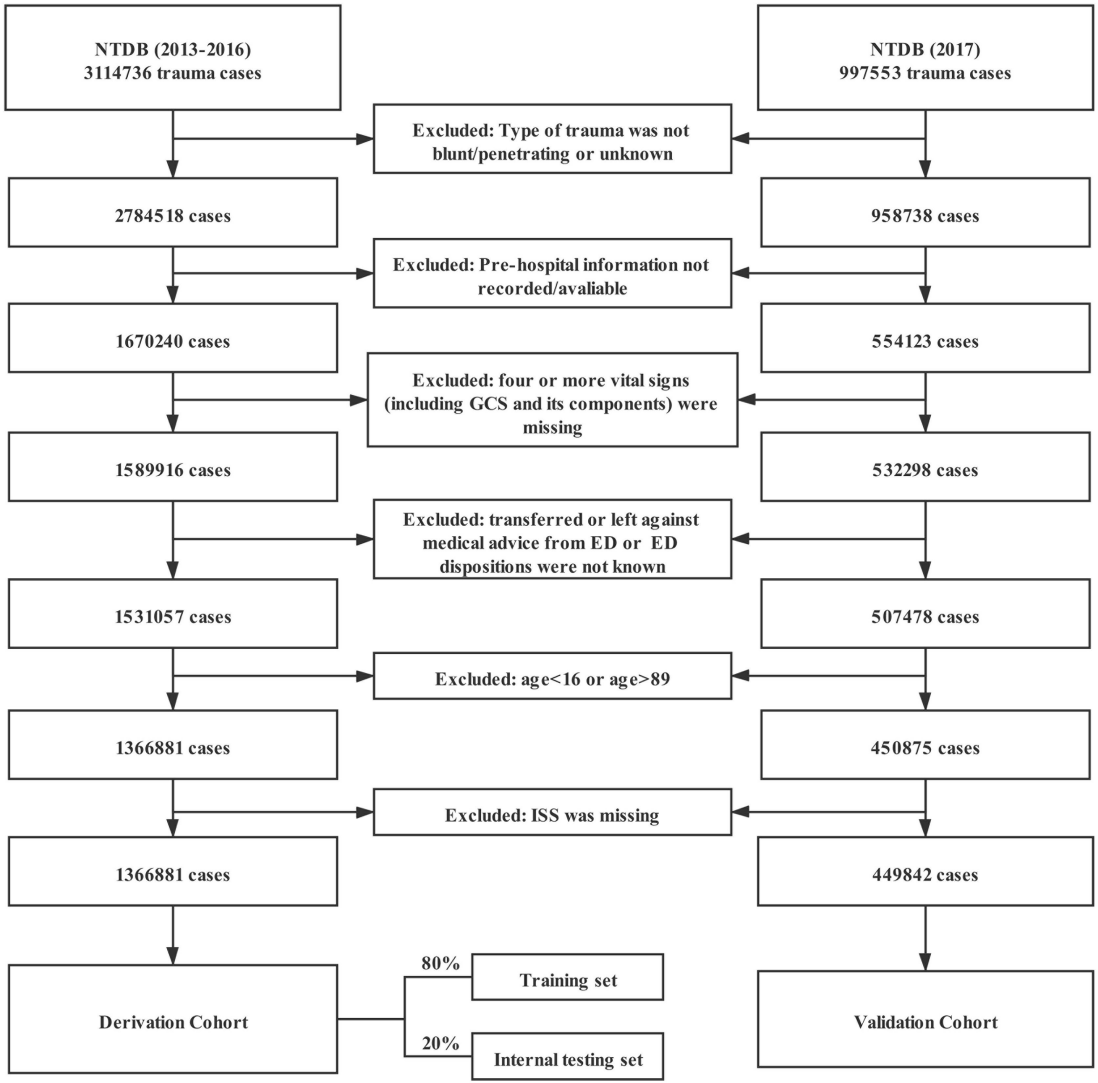
Study participant selection procedure. *NTDB*, National Trauma Data Bank; *GCS*, Glasgow Coma Scale; *ED*, emergency department; and *ISS*, Injury Severity Score.

This study used data from 2013 to 2017 in the NTDB, totaling 4,112,308 cases. The type of trauma was limited to blunt and penetrating. Cases without emergency medical service (EMS) data were excluded. To improve the quality of the included data, cases with more than three missing variables in the seven variables of SBP, HR, RR, GCS eye-opening response, GCS speech score, GCS motor score, and total GCS score were excluded. In addition, patients who were transferred from the emergency department (ED) to other hospitals, refused treatment in the ED, or had unknown outcomes in the ED were excluded. The age range of the patients was limited to 16 to 89 years (Figure 1).

### Measurements and Outcome

Cases from 2013 to 2016 were used as the derivation cohort, and cases from 2017 were used as the validation cohort. Eighty percent of the derivation cohort was randomly assigned to the training set, and the remaining 20% was used as the internal testing set. Predictor variables for the study included age and vital signs that were first recorded in the field. Whether the GCS motor was <6, i.e., whether the patient had the ability to follow commands, was a simplified assessment of consciousness used in this study as an alternative to the GCS. The outcome variable was in-hospital death from any cause. The missing values in the derivation or validation cohort were imputed using multivariate imputation by chained equations (MICE) (19). In addition to the study variables, vital signs recorded in the emergency department (ED), type of trauma, injury severity score (ISS), length of hospital stay, duration of mechanical ventilation, and length of intensive care unit (ICU) stay were also used for imputation. Due to the overlarge amount of data, only one imputed dataset was used for model development and validation.

### Analysis

In the training set, the neural network algorithm and logistic regression analysis were used to develop mortality prediction models in trauma patients. The neural network consists of an input layer, hidden layers, and an output layer, where the neurons in each layer are first activated by neurons in the previous layer, then transformed by a non-linear function in the current layer, and eventually input to the next layer (20). This non-linear characteristic makes it efficient at learning complex relationships of input variables. Three models were developed using three combinations of variables based on the neural network respectively. The first combination of variables was GCS, age, pulse rate, SBP, and peripheral oxygen saturation, referred to as “GAPSO.” The second combination of variables replaced GCS with a simpler binary assessment of GCS-Motor (GCS-m) score <6 (i.e., the ability to follow commands), referred to as “CAPSO.” The third combination removed SBP from the second combination to further investigate the effect of removing blood pressure on the model's performance, referred to as “CAPO.” The neural network models in this study contain two hidden layers with 256 and 128 neurons. They were optimized using the Adam optimizer, and overfitting was prevented by setting the dropout layer and early stopping.

Logistic regression analysis was performed, and then a score was developed using the second variable combinations, i.e., CAPSO. Considering the non-linear relationship between the variables and the outcome, the results of the multivariate generalized additive model were used to delineate the range of all predictors. Simple integers were assigned to the intervals according to the coefficients of the logistic regression, referring to the development of TRIAGES (8). Detailed methods for delineating variable intervals and assigning integer values are provided in the Supplementary Material. To be compared with the new models, the trauma scores previously developed were calibrated to the population in this study by fitting a logistic regression model to predict mortality for each score in the training set. Receiver operating characteristic (ROC) curves were plotted to compare the classification performance of each model as well as each score. The area under the receiver operating characteristic curves (AUROCs) were compared between different models using Delong's test (21). The agreement between the predicted probabilities of models or scores and observed frequencies of in-hospital mortality of trauma patients was assessed using probability calibration curves. To compare the sensitivity, specificity, and accuracy of the models and scores, it is necessary to select the threshold of mortality, whereby the prediction samples were classified into positive and negative samples. In this study, with reference to a previous study (5), the threshold with a sensitivity of at least 95% was set for comparison. Finally, to compare the differences between models when further classifying trauma patients, the trauma mortality predicted by each model and score was divided into three intervals as described in previous studies (5, 6): trauma patients at low (<5%), intermediate, and high (>50%) risk of death. The Shapley additive explanation (SHAP) plots (22) for the CAPSO model based on neural network were drawn. All statistical analyses were performed using Python (version 3.7.8) and R (version 4.0.2); neural network models were based on TensorFlow 2.1.0; *p* < 0.05 was considered statistically significant.

## Results

### Characteristics of Study Subjects

Based on the inclusion and exclusion criteria, a total of 1,816,723 cases were included in the study, with 1,366,881 cases in the derivation cohort and 449,842 cases in the validation cohort (Figure 1). The main characteristics of the trauma patients after imputation of missing values are shown in Table 1. The overall median age of all cases was 52 years, and the interquartile range (IQR) was 31 to 70 years. A total of 61.5% of patients were male, and the overall mortality rate was 4.8%. The baseline characteristics before imputation of missing values are shown in Supplementary Table 1.

**Table 1:** Baseline characteristics of trauma patients.

**Variables**	**Derivation cohort** **(***n*** = 1,366,881)**	**Validation cohort** **(***n*** = 449,842)**
Age, years [range]	52.0 [31.0, 70.0]	53.0 [32.0, 71.0]
Male, *n* (%)	843,371 (61.7)	273,196 (60.7)
Race, *n* (%)		
American Indian	11,196 (0.8)	3,492 (0.8)
Asian	25,923 (1.9)	9,285 (2.1)
Black or African American	204,017 (14.9)	68,516 (15.2)
Native Hawaiian or Other Pacific Islander	3,422 (0.3)	1,203 (0.3)
White	990,206 (72.4)	322,870 (71.8)
Other	132,117 (9.7)	44,476 (9.9)
Type of trauma, *n* (%)		
Blunt	1,230,013 (90.0)	404,176 (89.8)
Penetrating	136,868 (10.0)	45,666 (10.2)
First recorded vital signs measured at the scene of injury		
Systolic blood pressure, mmHg [range]	137.0 [120.0, 154.0]	138.0 [120.0, 156.0]
Pulse rate, beats/min [range]	89.0 [77.0, 102.0]	88.0 [76.0, 102.0]
Respiratory rate, rate/min [range]	18.0 [16.0, 20.0]	18.0 [16.0, 20.0]
Oxygen saturation, % [range]	98.0 [96.0, 99.0]	98.0 [95.0, 99.0]
Glasgow Coma Scale [range]	15.0 [14.0, 15.0]	15.0 [14.0, 15.0]
Injury Severity Score, [range]	9.0 [4.0, 13.0]	9.0 [4.0, 13.0]
Outcomes		
Length of stay in hospital, days [range]	4.0 [2.0, 7.0]	4.0 [2.0, 7.0]
ICU admission, *n* (%)	437,882 (32.0)	130,167 (28.9)
Mechanical ventilation, *n* (%)	209,471 (15.3)	55,315 (12.3)
Death, *n* (%)	65,770 (4.8)	22,208 (4.9)

*Medians with 25th−75th interquartile ranges are shown for continuous variables, and counts with percentages are shown for categorical variables*.

### Development of Mortality Prediction Models

Based on the neural network algorithm, three models were developed using three sets of variable combinations respectively, including neural network-based GAPSO (NN-GAPSO), neural network-based CAPSO (NN-CAPSO), and neural network-based CAPO (NN-CAPO). The continuous variables in the predictor combination CAPSO were classified into categorical variables based on the generalized additive model. After analysis through logistic regression, the new score, CAPSO (the Ability to Follow Commands, Age, Pulse Rate, Systolic Blood Pressure, and peripheral Oxygen saturation), was defined after assigning integer values to the variables according to the coefficients of the regression equation. The CAPSO scores ranged from a maximum of 18 to a minimum of 0, with higher scores representing higher risk of death. A score of five, the highest in one category, was assigned to the inability to follow commands. A score of four was assigned to systolic blood pressure between 0 and 49, which was the second-highest score in one category (Table 2).

**Table 2:** Predictors at presentation associated with in-hospital death used to develop CAPSO in the derivation dataset.

**Predictors**	**Beta [95% CI]**	***P* value**	**Integerized score point**
Intercept	−4.93 [−4.95, −4.90]	<0.001	
**Age, years**			
16–49	Reference		0
50–64	0.42 [0.39, 0.45]	<0.001	1
65–74	0.92 [0.88,0.95]	<0.001	2
75+	1.27 [1.24, 1.30]	<0.001	3
**Glasgow Coma Scale-Motor**			
<6	2.39 [2.37, 2.41]	<0.001	5
6	Reference		0
**Systolic blood pressure, mmHg**			
0–49	2.25 [2.19, 2.31]	<0.001	4
50–89	1.00 [0.87, 1.04]	<0.001	2
90–109	0.52 [0.49, 0.55]	<0.001	1
110–199	Reference		0
200+	0.54 [0.49, 0.59]	<0.001	1
**Pulse rate, beats/min**			
0–49	1.26 [1.21, 1.32]	<0.001	3
50–59	0.63 [0.58, 0.68]	<0.001	1
60–119	Reference		0
120–189	0.60 [0.57, 0.63]	<0.001	1
190+	1.39 [1.11, 1.66]	<0.001	3
**Oxygen saturation, %**			
0–79	1.50 [1.46, 1.54]	<0.001	3
80–89	0.95 [0.94, 0.98]	<0.001	2
90–94	0.41 [0.37, 0.44]	<0.001	1
95–100	Reference		0

*95% CI, 95% confidence interval; mmHg, millimeter of mercury*.

### Validation of the Models

**Figure 2:**
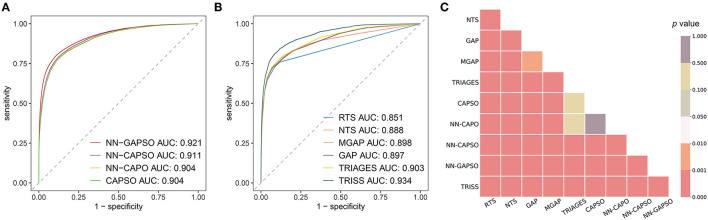
The discrimination of models/scores in the validation cohort. **(A)** Receiver operating characteristic curves for newly developed models; **(B)** receiver operating characteristic curves for trauma scores; **(C)** *p* values for a two-by-two comparison between different models and scores. *NN*, Neural network; *GAPSO*, Glasgow Coma Scale, Age, Pulse Rate, Systolic Blood Pressure, and Peripheral Oxygen saturation; *CAPSO*, the Ability to Follow Commands, Age, Pulse Rate, Systolic Blood Pressure, and Peripheral Oxygen saturation; *CAPO*, the Ability to Follow Commands, Age, Pulse Rate, and Peripheral Oxygen saturation; *RTS*, Revised Trauma Score; *NTS*, New Trauma Score; *MGAP*, Mechanism, Glasgow Coma Scale, Age, and Arterial Pressure; *GAP*, Glasgow Coma Scale, Age, and Systolic Blood Pressure score; *TRIAGES*, Trauma Rating Index in Age, Glasgow Coma Scale, Respiratory rate and Systolic blood pressure; *TRISS*, Trauma and Injury Severity Score.

**Figure 3:**
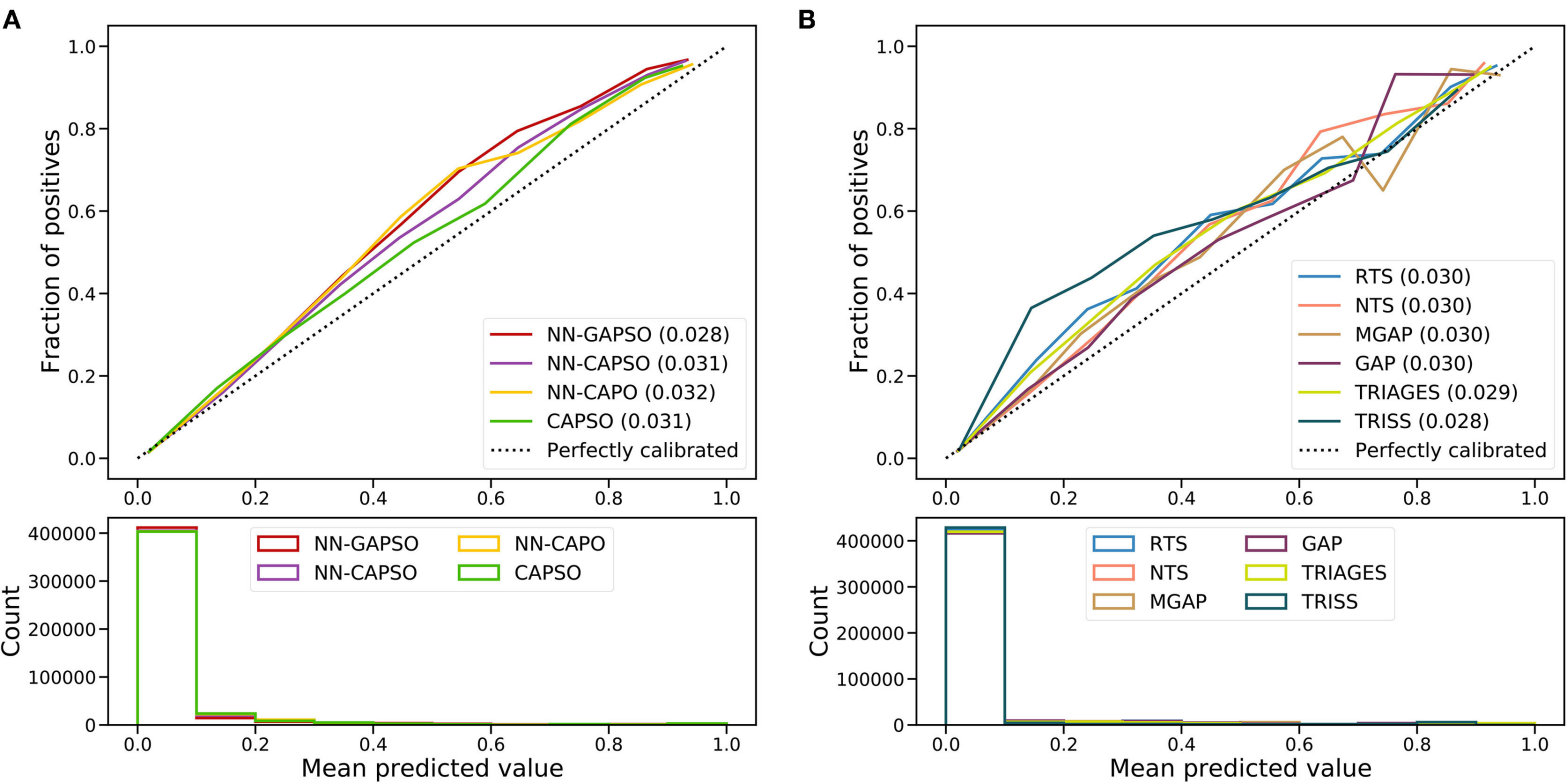
Calibration curves of newly developed models **(A)** and trauma scores **(B)** in the validation cohort. *NN*, Neural network; *GAPSO*, Glasgow Coma Scale, Age, Pulse Rate, Systolic Blood Pressure, and Peripheral Oxygen saturation; *CAPSO*, the Ability to Follow Commands, Age, Pulse Rate, Systolic Blood Pressure, and Peripheral Oxygen saturation; *CAPO*, the Ability to Follow Commands, Age, Pulse Rate, and Peripheral Oxygen saturation; *RTS*, Revised Trauma Score; *NTS*, New Trauma Score; *MGAP*, Mechanism, Glasgow Coma Scale, Age, and Arterial Pressure; *GAP*, Glasgow Coma Scale, Age, and Systolic Blood Pressure Score; *TRIAGES*, Trauma Rating Index in Age, Glasgow Coma Scale, Respiratory rate and Systolic blood pressure; *TRISS*, Trauma and Injury Severity Score.

The AUROC analysis showed that the neural network models had excellent performance in both the internal testing set and the validation set (internal testing set: Supplementary Table 2, Supplementary Figure 1; validation set: Table 3, Figure 2). NN-GAPSO showed the highest performance using the total GCS. NN-CAPSO replaced the initial GCS with the assessment of whether the GCS-m was <6, and its AUROC was lower than that of NN-GAPSO (*p* < 0.001). After further removal of systolic blood pressure, the AUROC values of NN-CAPO decreased in comparison to NN-CAPSO (*p* < 0.001). The AUROC of the CAPSO score was lower than that of NN-GAPSO and NN-CAPSO (both *p* < 0.001) but the same as that of NN-CAPO (*p* > 0.05). The AUROCs of NN-GAPSO and NN-CAPSO were higher than those of other scores (except TRISS), such as RTS, NTS, GAP, MGAP, and TRIGAGES (all *p* < 0.001). The AUROCs of NN-CAPO and CAPSO scores were similar to that of TRIAGES (both *p* > 0.05) and higher than the rest of the above scores (all *p* < 0.001). The sensitivity, specificity and accuracy of the models and scores according to the sensitivity closest to 0.95 are shown in Table 3. The probability calibration curves in the validation set of the neural network models, CAPSO score, and other trauma scores calibrated with the training set are shown in Figure 3, while those in the internal testing set are shown in Supplementary Figure 2. The SHAP plots of NN-CAPSO model are shown in Supplementary Figure 3.

**Table 3:** Comparison of the diagnostic properties of the models/scores at a sensitivity threshold of nearest 95%.

**Models/Scores**	**Variables**	**AUROC [95% CI]**	**Sensitivity [95% CI]**	**Specificity [95% CI]**	**Accuracy [95% CI]**
NN-GAPSO	GCS, Age, Pulse rate, SBP, SpO_2_	0.921 [0.918, 0.923]	0.951 [0.949, 0.954]	0.559 [0.557, 0.560]	0.578 [0.577, 0.580]
NN-CAPSO	Ability to follow commands, Age, Pulse rate, SBP, SpO_2_	0.911 [0.909, 0.913]	0.951 [0.948, 0.954]	0.546 [0.545, 0.548]	0.566 [0.565, 0.568]
NN-CAPO	Ability to follow commands, Age, Pulse rate, SpO_2_	0.904 [0.902, 0.906]	0.951 [0.948, 0.954]	0.518 [0.517, 0.520]	0.540 [0.538, 0.541]
CAPSO	Ability to follow commands, Age, Pulse rate, SBP, SpO_2_	0.904 [0.902, 0.906]	0.960 [0.957, 0.963]	0.492 [0.490, 0.493]	0.515 [0.513, 0.516]
RTS	SBP, RR, GCS	0.851 [0.848, 0.854]	0.760 [0.754, 0.765]	0.879 [0.878, 0.880]	0.873 [0.872, 0.874]
NTS	SBP, SpO_2_, GCS	0.888 [0.885, 0.891]	0.938 [0.935, 0.942]	0.391 [0.390, 0.393]	0.418 [0.417, 0.420]
MGAP	Mechanism, GCS, Age, SBP	0.898 [0.896, 0.901]	0.952 [0.949, 0.955]	0.451 [0.449, 0.452]	0.476 [0.474, 0.477]
GAP	GCS, Age, SBP	0.897 [0.894, 0.899]	0.967 [0.965, 0.970]	0.377 [0.375, 0.378]	0.406 [0.404, 0.407]
TRIAGES	GCS, Age, SBP, RR	0.903 [0.900, 0.905]	0.976 [0.974, 0.978]	0.349 [0.347, 0.350]	0.380 [0.378, 0.381]
TRISS	Mechanism, GCS, Age, SBP, RR, ISS	0.934 [0.932, 0.936]	0.959 [0.956, 0.961]	0.575 [0.573, 0.576]	0.594 [0.592, 0.595]

*CI, confidence interval; NN, Neural network; GAPSO, Glasgow Coma Scale, Age, Pulse Rate, Systolic Blood Pressure, and Peripheral Oxygen saturation; CAPSO, the Ability to Follow Commands, Age, Pulse Rate, Systolic Blood Pressure, and Peripheral Oxygen saturation; CAPO, the Ability to Follow Commands, Age, Pulse Rate, and Peripheral Oxygen saturation; RTS, Revised Trauma Score; NTS, New Trauma Score; MGAP, Mechanism, Glasgow Coma Scale, Age, and Arterial Pressure; GAP, Glasgow Coma Scale, Age, and Systolic Blood Pressure score; TRIAGES, Trauma Rating Index in Age, Glasgow Coma Scale, Respiratory rate and Systolic blood pressure; TRISS, Trauma and Injury Severity Score; AUROC, Area Under the Receiver Operating Characteristics; GCS, Glasgow Coma Scale; SBP, Systolic Blood Pressure; RR, Respiratory rate; SpO_2_, Peripheral Oxygen saturation; ISS, Injury Severity Score*.

Table 4 shows the reclassification of NN-CAPSO with NN-GAPSO, NN-CAPO, CAPSO score, TRIAGES score, and TRISS score in the validation set for the severity of trauma in 100,000 randomly selected patients. In NN-CAPSO, compared with NN-GAPSO, a total of 6,163 patients were misclassified out of 100,000 patients, of which 3,648 were overtriaged and the remaining 2,515 were undertriaged. Compared with NN-CAPSO, NN-CAPO misclassified 4,849 patients (overtriaged: 2,003, undertriaged: 2,846), while the CAPSO score misclassified 5,170 patients (overtriaged: 1,217, undertriaged: 3,953). When compared with TRIAGES, NN-CAPSO correctly reclassified 8,612 patients into the intermediate-risk group, which was classified by TRIAGES into the low-risk group, and correctly reclassified 344 patients into the high-risk group, which was classified by TRIAGES into the intermediate-risk group. However, in this comparison, NN-CAPSO incorrectly classified 326 patients who should have been in the high-risk group into the intermediate-risk group, and 927 patients who should have been in the intermediate-risk group were incorrectly classified into the low-risk group. Compared with TRISS, NN-CAPSO correctly classified 11,649 patients in the intermediate-risk group, who were classified as low risk by TRISS, but incorrectly classified 1,251 patients in the high-risk group as belonging to the intermediate-risk group.

**Table 4:** Reclassification of severity between NN-CAPSO and other scoring systems in the randomly selected validation cohort^a^.

**Reclassification of severity between NN-CAPSO and NN-GAPSO**					
		**NN-GAPSO**			
**Scores**	**Severity**	**Mild (<0.05 points)**	**Moderate (0.05 to 0.5 points)**	**Severe (>0.5 points)**	**Total**
NN-CAPSO	Mild (<0.05 points)	80,483 (0.96)	2,034 (6.64)	0 (0.0)	82,517 (1.1)
	Moderate (0.05 to 0.5 points)	3,500 (3.51)	11,921 (18.25)	481 (71.1)	15,902 (16.61)
	Severe (>0.5 points)	1 (0.0)	148 (36.49)	1,432 (90.92)	1,581 (85.77)
	Total	83,984 (1.06)	14,103 (16.77)	1,913 (85.94)	100,000 (4.9)
**Reclassification of severity between NN-CAPSO and NN-CAPO**					
		**NN-CAPO**			
**Scores**	**Severity**	**Mild (<0.05 points)**	**Moderate (0.05 to 0.5 points)**	**Severe (>0.5 points)**	**Total**
NN-CAPSO	Mild (<0.05 points)	80,675 (1.03)	1,842 (4.13)	0 (0.0)	82,517 (1.1)
	Moderate (0.05 to 0.5 points)	2,482 (6.08)	13,259 (18.06)	161 (59.63)	15,902 (16.61)
	Severe (>0.5 points)	0 (0.0)	364 (68.96)	1,217 (90.8)	1,581 (85.77)
	Total	83,157 (1.18)	15,465 (17.59)	1,378 (87.16)	100,000 (4.9)
**Reclassification of severity between NN-CAPSO and CAPSO**					
		**CAPSO**			
**Scores**	**Severity**	**Mild (<5 points)**	**Moderate (5 to 10 points)**	**Severe (>10 points)**	**Total**
NN-CAPSO	Mild (<0.05 points)	81,461 (1.06)	1,056 (4.17)	0 (0.0)	82,517 (1.1)
	Moderate (0.05 to 0.5 points)	3,708 (6.07)	12,033 (19.35)	161 (54.66)	15,902 (16.61)
	Severe (>0.5 points)	0 (0.0)	245 (67.35)	1336 (89.15)	1581 (85.77)
	Total	85,169 (1.28)	13,334 (19.03)	1,497 (85.44)	100,000 (4.9)
**Reclassification of severity between NN-CAPSO and TRIAGES**					
		**TRIAGES**			
**Scores**	**Severity**	**Mild (<4 points)**	**Moderate (5 to 8 points)**	**Severe (>9 points)**	**Total**
NN-CAPSO	Mild (<0.05 points)	81,590 (1.0)	927 (9.39)	0 (0.0)	82,517 (1.1)
	Moderate (0.05 to 0.5 points)	8,612 (6.08)	6,964 (27.31)	326 (65.95)	15,902 (16.61)
	Severe (>0.5 points)	18 (38.89)	344 (61.05)	1,219 (93.44)	1,581 (85.77)
	Total	90,220 (1.5)	8,235 (26.7)	1,545 (87.64)	100,000 (4.9)
**Reclassification of severity between NN-CAPSO and TRISS**					
		**TRISS**			
**Scores**	**Severity**	**Mild** **(>0.834 points)**	**Moderate (0.353 to 0.834 points)**	**Severe** **(<0.353 points)**	**Total**
NN-CAPSO	Mild (<0.05 points)	82,029 (0.98)	465 (20.22)	23 (34.78)	82,517 (1.1)
	Moderate (0.05 to 0.5 points)	11,649 (6.3)	3,002 (35.04)	1,251 (68.35)	15,902 (16.61)
	Severe (>0.5 points)	83 (36.14)	232 (68.53)	1,266 (92.18)	1,581 (85.77)
	Total	93,761 (1.67)	3,699 (35.28)	2,540 (79.92)	100,000 (4.9)

a *Data in parentheses are the percentages of deaths (%). Severe, high risk (> 50%) of death; Moderate, intermediate risk of death; Mild, low risk (<5%) of death. NN, Neural network; GAPSO, Glasgow Coma Scale, Age, Pulse Rate, Systolic Blood Pressure, and Peripheral Oxygen saturation; CAPSO, the Ability to Follow Commands, Age, Pulse Rate, Systolic Blood Pressure, and Peripheral Oxygen saturation; CAPO, the Ability to Follow Commands, Age, Pulse Rate, and Peripheral Oxygen saturation; RTS, Revised Trauma Score; NTS, New Trauma Score; MGAP, Mechanism, Glasgow Coma Scale, Age, and Arterial Pressure; GAP, Glasgow Coma Scale, Age, and Systolic Blood Pressure score; TRIAGES, Trauma Rating Index in Age, Glasgow Coma Scale, Respiratory rate and Systolic blood pressure; TRISS, Trauma and Injury Severity Score*.

## Discussion

The aim of this study was to develop a trauma mortality prediction model using a simple binary assessment of GCS-Motor (GCS-m) score <6, namely, whether the patient has the ability to follow commands, instead of the GCS. The prediction accuracy of the neural network-based CAPSO model was still higher than that of the other prehospital trauma scores using the total GCS, although it was slightly worse than that of the neural network-based GAPSO model, which uses the total GCS. In addition, the logistic regression-based CAPSO score had a predictive power similar to that of the TRIAGES score, and it was superior to other prehospital trauma scores. In addition, the neural network model NN-CAPO, which used the assessment of GCS-m <6 and removed the variable SBP, could achieve predictive accuracy similar to that of the TRIAGES.

In this study, the cutoff value for predicting the probability of death was chosen first based on the sensitivity closest to 95%, referring to a previous study (5). However, higher sensitivity tends to be accompanied by lower specificity, and in this dataset, even TRISS failed to reach the upper 60% of specificity. In addition, the reclassification table based on the classification of trauma patients according to minor, moderate, and severe injuries is more relevant for practical use. Furthermore, in this study, to make the results more intuitive, a random sample of 100,000 patients in the validation set was selected and grouped according to the 5% and 50% cutoff values of predicted mortality, referring to previous studies (5, 6). According to the reclassification table, NN-CAPSO could correctly triage more patients with moderate and severe injuries than the TRIAGES score.

In recent years, with the rise of machine learning algorithms, there has been an increasing number of studies using machine learning methods other than logistic regression algorithms to build prediction models. Most of these studies have suggested that machine learning algorithms have satisfying performance and broad application prospects in the medical field (23). Nevertheless, skepticism is also present. It was concluded that no evidence was found that the machine learning algorithms outperformed the logistic regression algorithm (24). In low-dimensional data, the machine learning algorithms were not considered to perform better than logistic regression (25). Some researchers have pointed out that the advantage of machine learning algorithms comes into play when dealing with data with a large number of features (26, 27), while others have claimed that machine learning algorithms require a larger data volume to demonstrate their performance (28). In addition to data quantity and dimensionality, the nature and processing of the features also play a very important role when comparing algorithms, such as the processing of continuous variables and the generation of interaction terms. In this study, although the number of features was relatively small, there was a sufficient amount of data, and the neural network models outperformed the logistic regression model and scores. Currently, with the widespread availability of smart electronic devices, machine learning models for predicting the outcomes of trauma patients, embedded into applications, will have higher accuracy and efficiency compared to scores calculated manually, whether applied for rapid assessment of trauma patient severity in normal times or in MCI. However, it is difficult to make machine learning algorithms interpretable, especially neural network algorithms, which are often referred to as “black boxes.” In this study, SHAP values are used to interpret the neural network model NN-CAPSO. Furthermore, machine learning algorithms are still not as commonly used in practice as intuitive scoring systems. Therefore, in addition to neural network algorithms, we have developed a simple scoring system for CAPSO using the logistic regression algorithm to facilitate the validation and use of this system in clinical settings.

Currently, pulse rate and peripheral oxygen saturation are easy to obtain in the prehospital phase. The accuracy of peripheral oxygen saturation measurement is relatively reliable when it is above 75% (29). In the CAPSO scoring system, we set the threshold for peripheral oxygen saturation at 80% according to the coefficients of the multivariate generalized additive model. In the SHAP plot, age was the second most important feature in the ranking. Age was also included as a variable in the MGAP, GAP, and TRIAGES scores which performed well in ROC analysis. By entering the age into the intelligent device in advance, it ensures that the age is available first when assessing the severity of the patient's injury by models or scores, which is applicable to people who wear the device earlier, such as military personnel or firefighters. However, if the patient is unconscious and the age is not available from all other sources in a short period of time, guesses by medical personnel can be useful but may lead to some degree of degradation in model accuracy, which is still subject to further validation. For the assessment of the patient's state of consciousness, the GCS is mostly used today. However, in specific situations, such as MCI, complete measurement of the GCS will waste precious time, as it has been reported that even formally trained clinicians have a probability of up to 20% of making errors in assessing GCS in a normal setting (30), let alone in the complicated trauma field. It was reported that the motor component of the GCS not only correlated linearly with survival but also retained most of the predictive validity of the GCS (31). When using GCS-m <6 as a predictor for the need for treatment at a trauma center, this predictor showed comparable validity to that using total GCS ≤ 13 (32). In addition, it was difficult to take manual measurements of blood pressure in the field (14). In this study, we attempted to replace the GCS with the ability to follow commands and further to remove SBP to build models and compare the effect of different models on the classification results. NN-GAPSO reached the highest AUROC as expected, while the performance of NN-CAPSO, NN-CAPO, and CAPSO deteriorated when compared to NN-GAPSO, but not as much as predicted. When the severity of the randomly selected patients was reclassified according to three intervals of mortality, the NN-GAPSO and NN-CAPSO disagreed on a total of 6,164 patients (6.164%, AUROC difference was nearly 0.01), while NN-CAPSO and CAPSO score had a different classification for a total of 5,170 patients (5.17%, AUROC difference was nearly 0.007). The slight sacrifice of models' performance in exchange for more ease of application was considered to make sense, even the accuracy of simplified models was not weaker than that of the other scores applicable to prehospital settings. The employment of the simpler model is estimated to increase user-friendliness and improve the efficiency of triage, although it remains to be evaluated in other datasets or in a real field setting. Furthermore, vital signs, assessment of consciousness, and age data of trauma patients may be missing due to specific comorbidities, injury conditions, or treatments. Despite the population with missing values is not very large, they may benefit from specific models developed for them. Alternatively, the use of algorithms that are able to handle missing values, or build models that treat missing values as special values, can preserve the information of the missing values themselves and facilitate the application to trauma patients with incomplete information, which requires further research.

In recent years, various emerging technologies are bringing about changes in the method of triage. The Wireless Vital Signs Monitor (WVSM) is a wireless vital sign monitoring device, and with its help, a health care worker can monitor up to 20 patients at the same time, making it very suitable for triaging in the field (33). Moreover, the use of smart glasses for remote classification is promising in reaching high accuracy, either through algorithms embedded into the glasses or by remote video connection to other physicians (34, 35). The use of wearable devices or radar for remote vital sign monitoring was supposed to save considerable manpower and time (36). However, it is complicated to apply GCS scores for consciousness assessment in the remote situation. In this case, the application of binary assessment of GCS-m score <6 rather than GCS would be effective. For example, some instructions from corresponding devices will ask the casualty to complete certain actions, and feedback can then be input into the devices to determine whether the person has the ability to follow commands. Then, the scoring model embedded in devices would give advice on triage. Furthermore, it is not easy to measure SBP with lightweight wearable devices or radar. Therefore, using machine learning algorithms to develop triage models not involving SBP will also be highly applicable now and in the near future.

In summary, the new user-friendly CAPSO system makes it possible to rapidly and reliably predict in-hospital mortality in trauma patients. It is suitable for future prehospital intelligent automated triage applications and is expected to improve the efficiency of triage by integration into prehospital decision-making systems.

## Limitations

This study used only data from the NTDB database and therefore has the limitations of that database. A portion of patients with much missing information were excluded, but the absence of vital signs may be due to the patient being agitated or receiving emergency medical care, etc., so the final study population included may have been to a degree biased, although the large sample population of this study is likely to be useful in reducing bias. While the data from 2017 were used separately for validation, the models or scores created for this study still require validation with data from other sources, particularly prospective data. For comparison with other studies, the cut-off values for the probability selected for this study referenced previous studies; however, in practice, the results of the model or score should be corrected and the appropriate cut-off values should be selected for decision making based on the application scenario.

## Data Availability Statement

The datasets analyzed for this study are available for purchase from the American College of Surgeons (ACS) via https://www.facs.org/quality-programs/trauma/tqp/center-programs/ntdb/datasets.

## Ethics Statement

All data provided by the NTDB are de-identified. Ethical review and approval was not required for the study on human participants in accordance with the local legislation and institutional requirements. Written informed consent for participation was not required for this study in accordance with the national legislation and the institutional requirements.

## Author Contributions

YLi designed the study, analyzed the data, and drafted the manuscript. LW and YLiu contributed to the acquisition of data and conducted data cleaning. YF, RY, and MY analyzed and interpreted the data. YZ, FZ, and HK jointly conceived of and designed this study. ZZ and HK conducted critical revision of the article. All of the authors reviewed and approved the final manuscript.

## Funding

This work was supported by the National Key Research and Development Program of China (2020YFB1313901) and Research and Development Project of Medical Big Data and Artificial Intelligence in PLA General Hospital (2019MBD-014).

## Conflict of Interest

The authors declare that the research was conducted in the absence of any commercial or financial relationships that could be construed as a potential conflict of interest.

## Publisher's Note

All claims expressed in this article are solely those of the authors and do not necessarily represent those of their affiliated organizations, or those of the publisher, the editors and the reviewers. Any product that may be evaluated in this article, or claim that may be made by its manufacturer, is not guaranteed or endorsed by the publisher.

## References

[1] Polinder S Haagsma JA Toet H Beeck E. Epidemiological burden of minor, major and fatal trauma in a national injury pyramid. Br J Surg. (2011) 99(S1):114–21. 10.1002/bjs.7708 22441864

[2] Wright CA. A National Evaluation of the Effect of Trauma-center Care on mortality. J Trauma Nurs. (2006) 13:366. 10.1097/00043860-200607000-00018 16436768

[3] Kluger Y Coccolini F Catena F Ansaloni L. WSES Handbook of Mass Casualties Incidents Management: Cham: Springer (2020).

[4] Champion HR Sacco WJ Copes WS Gann DS Gennarelli TA Flanagan ME. A revision of the Trauma Score. J Trauma. (1989) 29:623–9. 10.1097/00005373-198905000-00017 2657085

[5] Sartorius D Le Manach Y David JS Rancurel E Smail N Thicoïpé M. Mechanism, Glasgow Coma Scale, Age, and Arterial Pressure (MGAP): a new simple prehospital triage score to predict mortality in trauma patients. Crit Care Med. (2010) 38:831–7. 10.1097/CCM.0b013e3181cc4a67 20068467

[6] Kondo Y Abe T Kohshi K Tokuda Y Cook EF Kukita I. Revised trauma scoring system to predict in-hospital mortality in the emergency department: Glasgow coma scale, age, and systolic blood pressure score. Crit Care. (2011) 15:R191. 10.1186/cc10348 21831280 PMC3387633

[7] Jeong JH Park YJ Kim DH Kim TY Kang C Lee SH. The new trauma score (NTS): a modification of the revised trauma score for better trauma mortality prediction. BMC Surg. (2017) 17:77. 10.1186/s12893-017-0272-4 28673278 PMC5496419

[8] Shiraishi A Otomo Y Yoshikawa S Morishita K Roberts I Matsui H. Derivation and validation of an easy-to-compute trauma score that improves prognostication of mortality or the Trauma Rating Index in Age, Glasgow Coma Scale, Respiratory rate and Systolic blood pressure (TRIAGES) score. Crit Care. (2019) 23:365. 10.1186/s13054-019-2636-x 31752938 PMC6868841

[9] Baker SP. o'Neill B, Haddon W Jr, Long WB. The injury severity score: a method for describing patients with multiple injuries and evaluating emergency care. J Trauma Acute Care Surg. (1974) 14:187–96. 10.1097/00005373-197403000-00001 4814394

[10] Boyd CR Tolson MA Copes WS. Evaluating trauma care: the TRISS method. Trauma Score and the Injury Severity Score. J Trauma. (1987) 27:370–8. 10.1097/00005373-198704000-00005 3106646

[11] Reith FC Van den Brande R Synnot A Gruen R Maas AI. The reliability of the Glasgow Coma Scale: a systematic review. Intensive Care Med. (2016) 42:3–15. 10.1007/s00134-015-4124-3 26564211

[12] Riechers RG Ramage A Brown W Kalehua A Rhee P Ecklund JM. Physician knowledge of the Glasgow Coma Scale. J Neurotrauma. (2005) 22:1327–34. 10.1089/neu.2005.22.1327 16305321

[13] Chou R Totten AM Carney N Dandy S Fu R Grusing S. Predictive utility of the total Glasgow Coma Scale Versus the Motor Component of the Glasgow Coma Scale for Identification of patients with serious Traumatic injuries. Ann Emerg Med. (2017) 70:143–57.e6. 10.1016/j.annemergmed.2016.11.032 28089112

[14] McManus J Yershov AL Ludwig D Holcomb JB Salinas J Dubick MA. Radial pulse character relationships to systolic blood pressure andtrauma outcomes. Prehosp Emerg Care. (2005) 9:423–8. 10.1080/10903120500255891 16263676

[15] Churpek MM Yuen TC Winslow C Meltzer DO Kattan MW Edelson DP. Multicenter comparison of machine learning methods and conventional regression for predicting clinical deterioration on the wards. Crit Care Med. (2016) 44:368. 10.1097/CCM.0000000000001571 26771782 PMC4736499

[16] Kupas DF Melnychuk EM Young AJ. Glasgow Coma Scale motor component (“Patient Does Not Follow Commands”) performs similarly to total Glasgow Coma Scale in predicting severe injury in Trauma patients. Ann Emerg Med. (2016) 68:744–50.e3. 10.1016/j.annemergmed.2016.06.017 27436703

[17] Hashmi ZG Kaji AH Nathens AB. Practical guide to surgical data sets: National Trauma Data Bank (NTDB). JAMA Surg. (2018) 153:852–3. 10.1001/jamasurg.2018.0483 29617536

[18] Collins GS Reitsma JB Altman DG Moons KG. Transparent reporting of a multivariable prediction model for individual prognosis or diagnosis (TRIPOD): the TRIPOD statement. Br J Surg. (2015) 102:148–58. 10.1002/bjs.9736 25627261

[19] Buuren SV Groothuis-Oudshoorn K. MICE: Multivariate Imputation by Chained Equations in R. J Stat Softw. (2011) 45:1–67. 10.18637/jss.v045.i03

[20] Kriegeskorte N Golan T. Neural network models and deep learning. Current Biology. (2019) 29: R231–6. 10.1016/j.cub.2019.02.034 30939301

[21] Delong ER Delong DM Clarke-Pearson DL. Comparing the areas under two or more correlated receiver operating characteristic curves: a nonparametric approach. Biometrics. (1988) 44:837. 10.2307/2531595 3203132

[22] Lundberg SM Erion G Chen H DeGrave A Prutkin JM Nair B. From local explanations to global understanding with explainable AI for trees. Nat Mach Intell. (2020) 2:56–67. 10.1038/s42256-019-0138-9 32607472 PMC7326367

[23] Beam AL Kohane IS. Big data and machine learning in health care. JAMA. (2018) 319:1317–8. 10.1001/jama.2017.18391 29532063

[24] Ec A Jie MB Gscb C Ews D Jyvae F Bvca D. systematic review shows no performance benefit of machine learning over logistic regression for clinical prediction models. J Clin Epidemiol. (2019) 110:12–22. 10.1016/j.jclinepi.2019.02.004 30763612

[25] Gravesteijn BY Nieboer D Ercole A Lingsma HF Zoerle T. Machine learning algorithms performed no better than regression models for prognostication in traumatic brain injury. J Clin Epidemiol. (2020) 122:95–107. 10.1016/j.jclinepi.2020.03.005 32201256

[26] Deo RC Nallamothu BK. Learning about machine learning: the promise and pitfalls of big data and the electronic health record. Am Heart Assoc. (2016) 9:618–20. 10.1161/CIRCOUTCOMES.116.003308 28263936 PMC5832331

[27] Rajkomar A Oren E Chen K Dai AM Hajaj N Hardt M. Scalable and accurate deep learning for electronic health records. NPJ Digit Med. (2018) 1:18. 10.1038/s41746-018-0029-1 31304302 PMC6550175

[28] Tjeerd V Austin PC Steyerberg EW. Modern modelling techniques are data hungry: a simulation study for predicting dichotomous endpoints. BMC Med Res Methodol. (2014) 14:137. 10.1186/1471-2288-14-137 25532820 PMC4289553

[29] Wouters PF Gehring H Avgerinos J Konecny E Meyfroidt G. Accuracy of pulse oximeters: the European multi-center trial. Anesth Analg. (2002) 94:S13–S6. 11900030

[30] Holt AW Bury LK Bersten AD Skowronski GA Vedig AE. Prospective evaluation of residents and nurses as severity score data collectors. Crit Care Med. (1992) 20:1688–91. 10.1097/00003246-199212000-00015 1458947

[31] Healey C Osler TM Rogers FB Healey MA Glan Ce LG Kilgo PD. Improving the Glasgow Coma Scale score: motor score alone is a better predictor. J Trauma. (2003) 54:671–8. 10.1097/01.TA.0000058130.30490.5D 12707528

[32] Brown JB Forsythe RM Stassen NA Peitzman AB Gestring ML. Evidence-based improvement of the National Trauma Triage Protocol: the Glasgow Coma Scale versus Glasgow Coma Scale motor subscale. J Trauma Acute Care Surg. (2014) 77:101–2. 10.1097/TA.0000000000000280 24977762 PMC4620030

[33] Salinas J Nguyen R Darrah MI Kramer GA Cancio LC. Advanced monitoring and decision support for battlefield critical care environment. US Army Med Dep J. (2011):73–81. 21607909

[34] Follmann A Ohligs M Hochhausen N Beckers SK Rossaint R Czaplik M. Technical support by smart glasses during a mass casualty incident: a randomized controlled simulation trial on technically assisted triage and telemedical app use in disaster medicine. J Med Internet Res. (2019) 21:e11939. 10.2196/11939 30609988 PMC6682285

[35] Mccoy E Alrabah R Weichmann W Langdorf MI Lotfipour S. Feasibility of telesimulation and google glass for mass casualty triage education and training. West J Emerg Med. (2019) 20:512–9. 10.5811/westjem.2019.3.40805 31123554 PMC6526878

[36] Kim D You S So S Lee J Yook S Jang DP. A data-driven artificial intelligence model for remote triage in the prehospital environment. PLoS ONE. (2018) 13:e0206006. 10.1371/journal.pone.0206006 30352077 PMC6198975

