# Supplementary material for: Development and Validation of a Simplified Prehospital Triage Model Using Neural Network to Predict Mortality in Trauma Patients: The Ability to Follow Commands, Age, Pulse Rate, Systolic Blood Pressure and Peripheral Oxygen Saturation (CAPSO) Model
Source: Front Med (Lausanne). 2021 Dec 10;8:810195. doi: 10.3389/fmed.2021.810195 (PMC8709125; doi:10.3389/fmed.2021.810195)
Supplement: Supplementary file 1 [file Data_Sheet_1.docx]

Supplementary Material

**Detailed methods for CAPSO score developed**

The CAPSO score was developed based on logistic regression analysis with reference to the TRIAGES. R scripts and logs were attached below.

**Step 1. Initial partitioning**

Predictors were partitioned into intervals. Age was divided by 5 years interval (i.e. …, 25 to 29, 30 to 34, 35 to 39, …); Pulse rate was divided by 10 beats/min interval (i.e. …, 40 to 49, 50 to 59, 60 to 69, …); SBP was divided by 10mmHg interval ((i.e. ..., 90 to 99, 100 to 109, 110 to 119, …), and 0 to 49 mmHg was integrated into the 1 category referred as TRIAGES; and Peripheral Oxygen saturation was divided by 5% interval (i.e. …, 80 to 84, 85 to 89…).

**Step 2. Determination of initial reference category**

The regression coefficients of each categorized predictor were estimated by logistic regression analysis. The category with the least mortality in each predictor variable (age 20 to 24 years old, GCS-m == 6, pulse rate 70 to 80 /beats/min, systolic blood pressure 130 to 139 mmHg, and peripheral oxygen saturation 100%) was selected as the initial reference category.

**R script and log**

FUNC = glm(
# Study outcome was defined as in-hospital mortality
I(Expired==1)~
# AGE was initially partitioned for every 5 years
I(AGE.I==3)+I(AGE.I==5)+I(AGE.I==6)+I(AGE.I==7)+I(AGE.I==8)+I(AGE.I==9)+I(AGE.I==10)+I(AGE.I==11)+I(AGE.I==12)+I(AGE.I==13)+I(AGE.I==14)+I(AGE.I==15)+I(AGE.I==16)+I(AGE.I==17)+
# The GCS-m==6 was initially used as raw value
I(OC.I==0)+
# Systolic blood pressure (SBP) was initially partitioned for every 10 /mmHg
I(SBP.I %in% c(0:4))+I(SBP.I==5)+I(SBP.I==6)+I(SBP.I==7)+I(SBP.I==8)+I(SBP.I==9)+I(SBP.I==10)+I(SBP.I==11)+I(SBP.I==12)+I(SBP.I==14)+I(SBP.I==15)+I(SBP.I==16)+I(SBP.I==17)+I(SBP.I==18)+I(SBP.I==19)+I(SBP.I==20)+I(SBP.I==21)+I(SBP.I==22)+I(SBP.I==23)+I(SBP.I==24)+I(SBP.I>=25)+

# PULSE was initially partitioned for every 10 /beats/min
I(PULSE.I==0)+I(PULSE.I==1)+I(PULSE.I==2)+I(PULSE.I==3)+I(PULSE.I==4)+I(PULSE.I==5)+I(PULSE.I==6)+I(PULSE.I==8)+I(PULSE.I==9)+I(PULSE.I==10)+I(PULSE.I==11)+I(PULSE.I==12)+I(PULSE.I==13)+I(PULSE.I==14)+I(PULSE.I==15)+I(PULSE.I==16)+I(PULSE.I==17)+I(PULSE.I==18)+I(PULSE.I>=19)+

# Peripheral Oxygen saturation was initially partitioned for every 5%
I(OXYSAT.I==0)+I(OXYSAT.I==1)+I(OXYSAT.I==2)+I(OXYSAT.I==3)+I(OXYSAT.I==4)+I(OXYSAT.I==5)+I(OXYSAT.I==6)+I(OXYSAT.I==7)+I(OXYSAT.I==8)+I(OXYSAT.I==9)+I(OXYSAT.I==10)+I(OXYSAT.I==11)+I(OXYSAT.I==12)+I(OXYSAT.I==13)+I(OXYSAT.I==14)+I(OXYSAT.I==15)+I(OXYSAT.I==16)+I(OXYSAT.I==17)+I(OXYSAT.I==18)+I(OXYSAT.I==19)
,
# Using logit binomial function as a link function.
family=binomial(link="logit"),
data=df)

summary(FUNC)

## Estimate Std. Error z value Pr(>|z|)
## (Intercept) -5.045124 0.031636 -159.472 < 2e-16 ***
## I(AGE.I == 3)TRUE 0.035204 0.031546 1.116 0.264443
## I(AGE.I == 5)TRUE 0.082500 0.027833 2.964 0.003036 **
## I(AGE.I == 6)TRUE 0.072249 0.029370 2.460 0.013895 *
## I(AGE.I == 7)TRUE 0.109119 0.031120 3.506 0.000454 ***
## I(AGE.I == 8)TRUE 0.097025 0.031682 3.062 0.002195 **
## I(AGE.I == 9)TRUE 0.100680 0.030866 3.262 0.001107 **
## I(AGE.I == 10)TRUE 0.314811 0.028572 11.018 < 2e-16 ***
## I(AGE.I == 11)TRUE 0.495687 0.027958 17.730 < 2e-16 ***
## I(AGE.I == 12)TRUE 0.701347 0.028183 24.886 < 2e-16 ***
## I(AGE.I == 13)TRUE 0.906618 0.028449 31.868 < 2e-16 ***
## I(AGE.I == 14)TRUE 1.098918 0.028474 38.594 < 2e-16 ***
## I(AGE.I == 15)TRUE 1.282894 0.027709 46.299 < 2e-16 ***
## I(AGE.I == 16)TRUE 1.345936 0.026990 49.868 < 2e-16 ***
## I(AGE.I == 17)TRUE 1.425724 0.026798 53.202 < 2e-16 ***
## I(OC.I == 0)TRUE 2.364516 0.011566 204.429 < 2e-16 ***
## I(SBP.I %in% c(0:4))TRUE 2.235378 0.036929 60.532 < 2e-16 ***
## I(SBP.I == 5)TRUE 1.310752 0.072221 18.149 < 2e-16 ***
## I(SBP.I == 6)TRUE 1.348367 0.043214 31.202 < 2e-16 ***
## I(SBP.I == 7)TRUE 1.050779 0.036572 28.732 < 2e-16 ***
## I(SBP.I == 8)TRUE 0.961740 0.029497 32.604 < 2e-16 ***
## I(SBP.I == 9)TRUE 0.709999 0.026665 26.627 < 2e-16 ***
## I(SBP.I == 10)TRUE 0.507812 0.025123 20.213 < 2e-16 ***
## I(SBP.I == 11)TRUE 0.265138 0.023386 11.338 < 2e-16 ***
## I(SBP.I == 12)TRUE 0.049160 0.023272 2.112 0.034650 *
## I(SBP.I == 14)TRUE 0.015491 0.022438 0.690 0.489941
## I(SBP.I == 15)TRUE 0.059124 0.024266 2.436 0.014832 *
## I(SBP.I == 16)TRUE -0.001763 0.026597 -0.066 0.947164
## I(SBP.I == 17)TRUE 0.129032 0.028985 4.452 8.52e-06 ***
## I(SBP.I == 18)TRUE 0.240299 0.030459 7.889 3.04e-15 ***
## I(SBP.I == 19)TRUE 0.268675 0.038209 7.032 2.04e-12 ***
## I(SBP.I == 20)TRUE 0.542408 0.042133 12.874 < 2e-16 ***
## I(SBP.I == 21)TRUE 0.577719 0.051594 11.197 < 2e-16 ***
## I(SBP.I == 22)TRUE 0.564803 0.062212 9.079 < 2e-16 ***
## I(SBP.I == 23)TRUE 0.786393 0.085398 9.209 < 2e-16 ***
## I(SBP.I == 24)TRUE 0.720154 0.107242 6.715 1.88e-11 ***
## I(SBP.I >= 25)TRUE 1.024255 0.109711 9.336 < 2e-16 ***
## I(PULSE.I == 0)TRUE 1.553926 0.048585 31.983 < 2e-16 ***
## I(PULSE.I == 1)TRUE 0.308529 0.153980 2.004 0.045103 *
## I(PULSE.I == 2)TRUE 1.379766 0.107251 12.865 < 2e-16 ***
## I(PULSE.I == 3)TRUE 1.550012 0.077870 19.905 < 2e-16 ***
## I(PULSE.I == 4)TRUE 1.145051 0.043435 26.362 < 2e-16 ***
## I(PULSE.I == 5)TRUE 0.669973 0.028341 23.640 < 2e-16 ***
## I(PULSE.I == 6)TRUE 0.261364 0.021675 12.058 < 2e-16 ***
## I(PULSE.I == 8)TRUE -0.056439 0.019745 -2.858 0.004257 **
## I(PULSE.I == 9)TRUE -0.061942 0.020978 -2.953 0.003150 **
## I(PULSE.I == 10)TRUE 0.088440 0.021778 4.061 4.89e-05 ***
## I(PULSE.I == 11)TRUE 0.210957 0.023622 8.931 < 2e-16 ***
## I(PULSE.I == 12)TRUE 0.465041 0.025102 18.526 < 2e-16 ***
## I(PULSE.I == 13)TRUE 0.725939 0.029570 24.550 < 2e-16 ***
## I(PULSE.I == 14)TRUE 0.853794 0.035254 24.219 < 2e-16 ***
## I(PULSE.I == 15)TRUE 0.921495 0.047479 19.409 < 2e-16 ***
## I(PULSE.I == 16)TRUE 1.043580 0.064490 16.182 < 2e-16 ***
## I(PULSE.I == 17)TRUE 1.010285 0.099676 10.136 < 2e-16 ***
## I(PULSE.I == 18)TRUE 1.044182 0.132348 7.890 3.03e-15 ***
## I(PULSE.I >= 19)TRUE 1.433386 0.140464 10.205 < 2e-16 ***
## I(OXYSAT.I == 0)TRUE 1.439371 0.045148 31.881 < 2e-16 ***
## I(OXYSAT.I == 1)TRUE 1.010502 0.174630 5.787 7.19e-09 ***
## I(OXYSAT.I == 2)TRUE 1.094765 0.187831 5.828 5.59e-09 ***
## I(OXYSAT.I == 3)TRUE 0.854809 0.181227 4.717 2.40e-06 ***
## I(OXYSAT.I == 4)TRUE 1.234963 0.174351 7.083 1.41e-12 ***
## I(OXYSAT.I == 5)TRUE 1.260838 0.274968 4.585 4.53e-06 ***
## I(OXYSAT.I == 6)TRUE 1.634305 0.194387 8.407 < 2e-16 ***
## I(OXYSAT.I == 7)TRUE 1.543424 0.197563 7.812 5.62e-15 ***
## I(OXYSAT.I == 8)TRUE 1.331038 0.167495 7.947 1.92e-15 ***
## I(OXYSAT.I == 9)TRUE 1.469089 0.163620 8.979 < 2e-16 ***
## I(OXYSAT.I == 10)TRUE 1.701470 0.091145 18.668 < 2e-16 ***
## I(OXYSAT.I == 11)TRUE 1.642956 0.104151 15.775 < 2e-16 ***
## I(OXYSAT.I == 12)TRUE 1.500063 0.076193 19.688 < 2e-16 ***
## I(OXYSAT.I == 13)TRUE 1.566383 0.067286 23.279 < 2e-16 ***
## I(OXYSAT.I == 14)TRUE 1.374383 0.048077 28.587 < 2e-16 ***
## I(OXYSAT.I == 15)TRUE 1.287254 0.039281 32.771 < 2e-16 ***
## I(OXYSAT.I == 16)TRUE 1.066424 0.029954 35.602 < 2e-16 ***
## I(OXYSAT.I == 17)TRUE 0.751356 0.025021 30.029 < 2e-16 ***
## I(OXYSAT.I == 18)TRUE 0.335921 0.019067 17.618 < 2e-16 ***
## I(OXYSAT.I == 19)TRUE -0.087126 0.016211 -5.375 7.68e-08 ***
## ---
## Signif. codes: 0 '***' 0.001 '**' 0.01 '*' 0.05 '.' 0.1 ' ' 1

**Step 3. Integration of adjunct categories**

Intervals with similar regression coefficients were integrated and logistic regression was repeated until the difference in regression coefficients between adjacent intervals was significant.

# Categories for the 7th (35 to 39 years old), 8th (40 to 44 years old), and

# 9th (45 to 49 years old) age category were integrated into 1 category. And

# categories for the 16th and 17th age categories were integrated.

## Estimate Std. Error z value Pr(>|z|)
## (Intercept) -5.05515 0.01543 -327.571 < 2e-16 ***
## I(AGE.I %in% c(7, 8, 9))TRUE 0.05379 0.01781 3.020 0.00253 **
## I(AGE.I == 10)TRUE 0.26406 0.02369 11.146 < 2e-16 ***
## I(AGE.I == 11)TRUE 0.44197 0.02293 19.274 < 2e-16 ***
## I(AGE.I == 12)TRUE 0.64722 0.02318 27.923 < 2e-16 ***
## I(AGE.I == 13)TRUE 0.85100 0.02346 36.270 < 2e-16 ***
## I(AGE.I == 14)TRUE 1.04091 0.02347 44.359 < 2e-16 ***
## I(AGE.I == 15)TRUE 1.22396 0.02248 54.441 < 2e-16 ***
## I(AGE.I %in% c(16, 17))TRUE 1.32542 0.01768 74.982 < 2e-16 ***
## I(OC.I == 0)TRUE 2.37151 0.01154 205.433 < 2e-16 ***

[…]

# Categories for 10th (50 to 54 years old) and 11th (55 to 59 years old)

# age category were integrated into 1 category. And categories for the 15-17th

# age category were integrated.

## Estimate Std. Error z value Pr(>|z|)
## (Intercept) -5.02936 0.01373 -366.194 <2e-16 ***
## I(AGE.I %in% c(10, 11))TRUE 0.33500 0.01697 19.740 <2e-16 ***
## I(AGE.I == 12)TRUE 0.62881 0.02217 28.369 <2e-16 ***
## I(AGE.I == 13)TRUE 0.83145 0.02245 37.032 <2e-16 ***
## I(AGE.I == 14)TRUE 1.02054 0.02243 45.492 <2e-16 ***
## I(AGE.I %in% c(15, 16, 17))TRUE 1.27603 0.01475 86.493 <2e-16 ***
## I(OC.I == 0)TRUE 2.37557 0.01152 206.191 <2e-16 ***
[…]

**Step 4. Assign integer values to variable categories based on regression coefficients**

Based on the coefficients of the logistic regression equation after combining the intervals, we chose 0.5 as the base to assign integer scores to each interval.

**Supplementary Table 1.**  Baseline characteristics of trauma patients before imputation of missing values.

| Variables | Missing | Derivation cohort (*n* = 1366881) | Validation cohort (*n* = 449842) |
| --- | --- | --- | --- |
| Age, years [range] |  | 51.0 [31.0,70.0] | 53.0 [32.0,71.0] |
| Male, *n* (%) |  | 843371 (61.7) | 273196 (60.7) |
| Race, *n* (%) | | | |
| American Indian |  | 11196 (0.8) | 3492 (0.8) |
| Asian |  | 25923 (1.9) | 9285 (2.1) |
| Black or African American |  | 204017 (14.9) | 68516 (15.2) |
| Native Hawaiian or Other Pacific Islander |  | 3422 (0.3) | 1203 (0.3) |
| White |  | 990206 (72.4) | 322870 (71.8) |
| Other |  | 132117 (9.7) | 44476 (9.9) |
| Type of trauma, *n* (%) |  |  |  |
| Blunt |  | 1230013 (90.0) | 404176 (89.8) |
| Penetrating |  | 136868 (10.0) | 45666 (10.2) |
| First recorded vital signs measured at the scene of injury | | | |
| Systolic blood pressure, mmHg [range] | 65897 | 137.0 [120.0,154.0] | 138.0 [121.0,156.0] |
| Pulse rate, beats/min [range] | 44170 | 89.0 [77.0,102.0] | 88.0 [76.0,102.0] |
| Respiratory rate, rate/min [range] | 83856 | 18.0 [16.0,20.0] | 18.0 [16.0,20.0] |
| Peripheral Oxygen saturation, % [range] | 389108 | 98.0 [96.0,99.0] | 98.0 [95.0,99.0] |
| Glasgow Coma Scale [range] | 2009 | 15.0 [14.0,15.0] | 15.0 [14.0,15.0] |
| Injury Severity Score, [range] |  | 9.0 [4.0,13.0] | 9.0 [4.0,13.0] |
| Outcomes | | | |
| Length of stay in hospital, days [range] | 20220 | 4.0 [2.0,7.0] | 4.0 [2.0,7.0] |
| ICU admission, *n* (%) |  | 437882 (32.0) | 130167 (28.9) |
| Mechanical ventilation, *n* (%) |  | 209471 (15.3) | 55315 (12.3) |
| Death, *n* (%) |  | 65770 (4.8) | 22208 (4.9) |

Medians with 25th–75th interquartile ranges are shown for continuous variables, and counts with percentages are shown for categorical variables.

**Supplementary Table 2.** Comparison of the diagnostic properties of the models/scores at a sensitivity threshold of 95% (or the nearest possible value) in the internal testing set

| Models/Scores | Variables | AUROC | Sensitivity | Specificity | Accuracy |
| --- | --- | --- | --- | --- | --- |
| NN-GAPSO | GCS, Age, Pulse rate, SBP, SpO_2_ | 0.896 [0.893, 0.899] | 0.951 [0.947,0.955] | 0.489 [0.487,0.491] | 0.512 [0.510,0.513] |
| NN-CAPSO | Ability to follow commands, Age, Pulse rate, SBP, SpO_2_ | 0.887 [0.884, 0.890] | 0.950 [0.946,0.954] | 0.483 [0.481,0.485] | 0.505 [0.503,0.507] |
| NN-CAPO | Ability to follow commands, Age, Pulse rate, SpO_2_ | 0.878 [0.875, 0.882] | 0.950 [0.947,0.954] | 0.452 [0.450,0.454] | 0.476 [0.474,0.478] |
| CAPSO | Ability to follow commands, Age, Pulse rate, SBP, SpO_2_ | 0.878 [0.875, 0.881] | 0.941 [0.937,0.945] | 0.507 [0.505,0.509] | 0.528 [0.526,0.530] |
| RTS | SBP, RR, GCS | 0.822 [0.817, 0.826] | 0.715 [0.708,0.723] | 0.865 [0.864,0.866] | 0.858 [0.856,0.859] |
| NTS | SBP, SpO_2_, GCS | 0.855 [0.851, 0.859] | 0.916 [0.912,0.921] | 0.397 [0.396,0.399] | 0.422 [0.421,0.424] |
| MGAP | Mechanism, GCS, Age, SBP | 0.870 [0.866, 0.873] | 0.939 [0.935,0.943] | 0.444 [0.442,0.446] | 0.468 [0.466,0.469] |
| GAP | GCS, Age, SBP | 0.870 [0.866, 0.873] | 0.952 [0.949,0.956] | 0.384 [0.382,0.386] | 0.411 [0.409,0.413] |
| TRIAGES | GCS, Age, SBP, RR | 0.877 [0.874, 0.881] | 0.965 [0.962,0.968] | 0.361 [0.359,0.363] | 0.390 [0.388,0.392] |
| TRISS | Mechanism, GCS, Age, SBP, RR, ISS | 0.910 [0.908, 0.913] | 0.957 [0.954,0.961] | 0.511 [0.509,0.512] | 0.532 [0.530,0.534] |

*NN* Neural network; *GAPSO* Glasgow Coma Scale, Age, Pulse Rate, Systolic Blood Pressure, and Peripheral Oxygen saturation; *CAPSO* the Ability to Follow Commands, Age, Pulse Rate, Systolic Blood Pressure, and Peripheral Oxygen saturation; *CAPO* the Ability to Follow Commands, Age, Pulse Rate, and Peripheral Oxygen saturation; *RTS* Revised Trauma Score; *NTS* New Trauma Score; *MGAP* Mechanism, Glasgow Coma Scale, Age, and Arterial Pressure; *GAP* Glasgow Coma Scale, Age, and Systolic Blood Pressure score; *TRIAGES* Trauma Rating Index in Age, Glasgow Coma Scale, Respiratory rate and Systolic blood pressure; *TRISS* Trauma and Injury Severity Score; *AUROC* Area Under the Receiver Operating Characteristics; *GCS* Glasgow Coma Scale; *SBP* Systolic Blood Pressure; *SpO_2_* Peripheral Oxygen saturation; *ISS* Injury Severity Score.

**
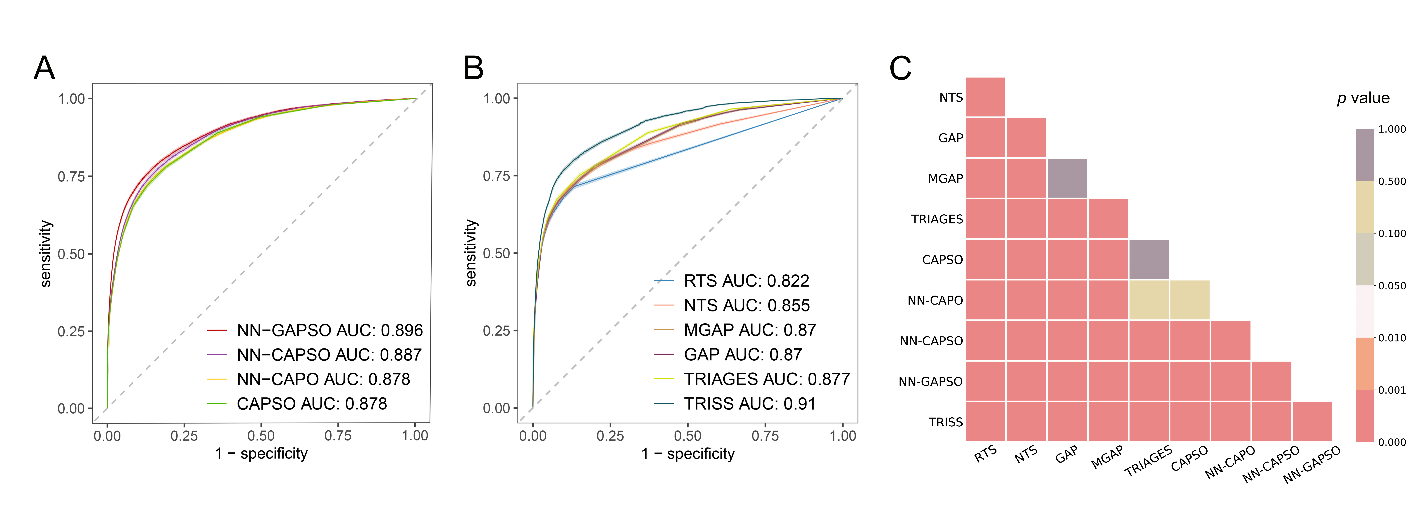
**

**Supplementary Figure 1.** **The discrimination of models/scores in the internal testing set.** (A) Receiver operating characteristic curves for newly developed models; (B) receiver operating characteristic curves for trauma scores; (C) p values for a two-by-two comparison between different models and scores. *NN* Neural network; *GAPSO* Glasgow Coma Scale, Age, Pulse Rate, Systolic Blood Pressure, and Peripheral Oxygen saturation; *CAPSO* the Ability to Follow Commands, Age, Pulse Rate, Systolic Blood Pressure, and Peripheral Oxygen saturation; *CAPO* the Ability to Follow Commands, Age, Pulse Rate, and Peripheral Oxygen saturation; *RTS* Revised Trauma Score; *NTS* New Trauma Score; *MGAP* Mechanism, Glasgow Coma Scale, Age, and Arterial Pressure; *GAP* Glasgow Coma Scale, Age, and Systolic Blood Pressure score; *TRIAGES* Trauma Rating Index in Age, Glasgow Coma Scale, Respiratory rate and Systolic blood pressure; *TRISS* Trauma and Injury Severity Score

**
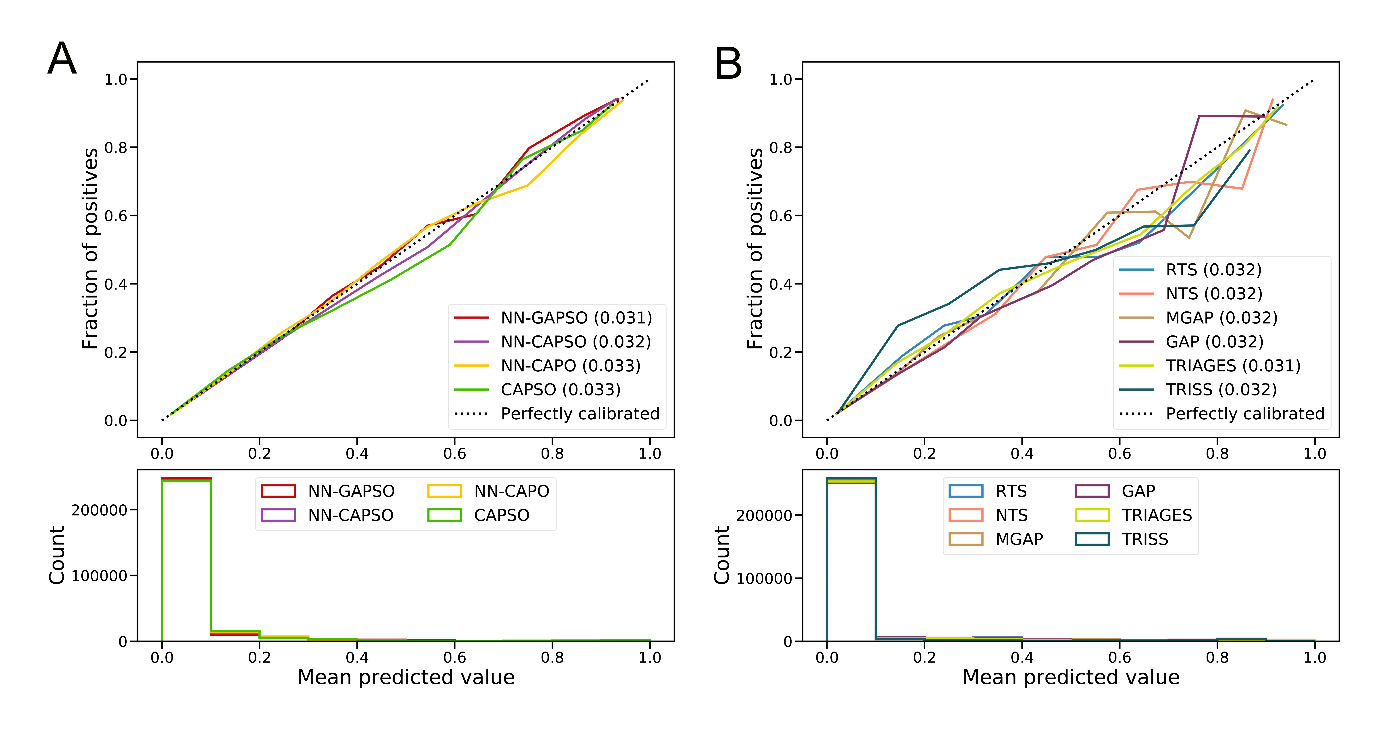
**

**Supplementary Figure 2. Calibration curves of newly developed models (A) and trauma scores (B) in the internal testing set**. *NN* Neural network; *GAPSO* Glasgow Coma Scale, Age, Pulse, Systolic Blood Pressure, and Peripheral Oxygen saturation; CAPSO the Ability to Follow Commands, Age, Pulse Rate, Systolic Blood Pressure, and Peripheral Oxygen saturation; CAPO the Ability to Follow Commands, Age, Pulse Rate, and Peripheral Oxygen saturation; *RTS* Revised Trauma Score; *NTS* New Trauma Score; *MGAP* Mechanism, Glasgow Coma Scale, Age, and Arterial Pressure; *GAP* Glasgow Coma Scale, Age, and Systolic Blood Pressure score; *TRIAGES* Trauma Rating Index in Age, Glasgow Coma Scale, Respiratory rate and Systolic blood pressure; *TRISS* Trauma and Injury Severity Score

**
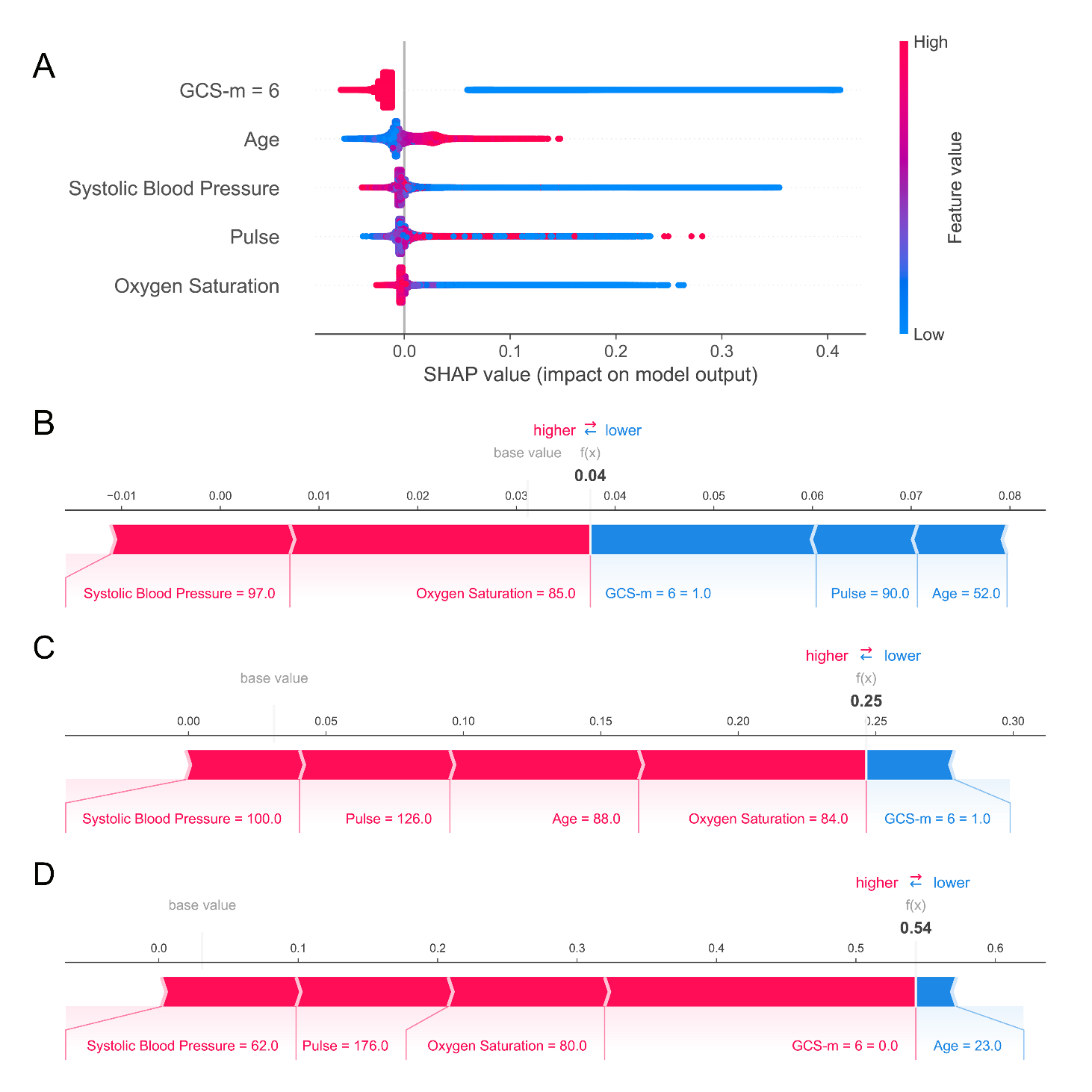
**

**Supplementary Figure 3. Explanation of the NN-CAPSO model by the SHapley Additive exPlanations (SHAP) values on the validation set. (A)** The impact of features on the model output. The color represents the value of the feature. The horizontal coordinates indicate positive and negative correlations with predicted mortality risk, with positive values indicating a positive effect on predicting mortality risk and negative values the opposite. (B-D) Explanation of the impact of features on prediction results of specific instances. Output values (bold) represent the predicted mortality of the patients. The features in red have positive effects on the output of higher mortality risk, while features in blue have the opposite effect. The interpretation of a low-risk instance (B), an intermediate-risk instance (C) and a high-risk instance (D) were showed respectively.
